# Returning home and re-negotiating mental health: a qualitative study of help-seeking, stigma, and cultural identity among Algerian international student returnees

**DOI:** 10.1080/17482631.2026.2724187

**Published:** 2026-08-26

**Authors:** Nesrine Boussaoui, Stephen Kelly, Nicola Cogan

**Affiliations:** a Department of Psychological Sciences and Health, University of Strathclyde, Glasgow, UK

**Keywords:** International students, mental health literacy, help-seeking, qualitative research, stigma, cultural identity

## Abstract

**Purpose:**

International students navigate major academic, social, and cultural transitions while studying abroad. However, less is known about their mental health after returning home. This qualitative study explored how Algerian international student returnees understood and navigated mental health, stigma, help-seeking, and coping after returning from the United Kingdom.

**Methods:**

Semi-structured interviews with Algerian returnees were analysed using reflexive thematic analysis within an interpretivist framework.

**Results:**

Reintegration was an emotionally complex transition shaped by cultural expectations, language, stigma, and evolving identity. Five interconnected themes were generated: (1) shifting perceptions and evolving understandings of mental health; (2) struggles of return and reverse culture shock; (3) silent struggles and barriers to help-seeking; (4) coping with the unfamiliar in familiar surroundings; and (5) linguistic duality in mental health narratives. Participants experienced uncertainty in interpreting and communicating distress, and limited confidence discussing mental health with family, peers, or professionals. Despite these challenges, they demonstrated resilience, adaptability, and coping strategies informed by their international experiences.

**Conclusion:**

Returning home represents a critical yet under-recognised period for mental health and wellbeing. Culturally responsive reintegration support, including accessible help-seeking pathways and re-entry programmes are needed to support the wellbeing across the full cycle of international students mobility.

## Introduction

International student mental health has received increasing scholarly attention, particularly in relation to adjustment difficulties during study abroad (Ansari Lari et al., 2025; Mahmood et al., 2026). However, far less is known about what happens after students return home. Research has predominantly focused on acculturation stress in host countries, leaving the psychological and identity-related consequences of reintegration comparatively underexplored. This omission is particularly significant for students from collectivist and religiously embedded societies, where understandings of mental health may differ substantially from Western psychological frameworks.

Research examining international student mental health has been heavily concentrated in Western host countries and has most frequently focused on students from East and South Asian contexts (Cogan et al., 2024; Dombou et al., 2023; Lee, 2026; Ma et al., 2020). In contrast, students from North African and Arab-majority countries remain significantly under-represented in the literature. Algeria provides an important context for understanding mental health following international study. Strong collectivist values, close family interdependence, and the central role of religion shape how emotional distress is understood, communicated, and managed (Mansouri, 2025). Mental health difficulties may be interpreted through moral, spiritual, or social frameworks rather than biomedical or psychological models, while help-seeking may be constrained by concerns about stigma, reputation, and family honour (Elshamy et al., 2023). Consequently, students returning with exposure to Western psychological discourse may experience tensions when renegotiating identity and wellbeing within their home cultural context. Examining Algerian international students (AISs) therefore extends the global mental health literature by bringing forward perspectives that remain underrepresented.

AIS studying in the United Kingdom encounter new discourses of therapy, self-expression, and mental health awareness. Prior findings from this broader project (Boussaoui et al., 2024) demonstrated that while abroad, many AISs interpreted distress through cultural and religious lenses, often hesitating to engage with formal services because of perceived stigma and cultural mismatch. Although exposure to Western psychological language expanded their conceptual repertoire, interpretations of distress remained embedded in collective, moral, and spiritual meanings (Rawdin & Dhillon, 2025). The present study extends these findings by examining how these understandings are renegotiated after students return to Algeria, with particular attention to reintegration, identity, help-seeking, and coping.

Reintegration is not simply a reversal of acculturation. Returning students must renegotiate identity within a context that is culturally familiar yet subjectively experienced as different following their time abroad. Berry's (1997) acculturation model explains how people navigating between cultures may experience tension between adopting new cultural norms and maintaining those of their home context. A similar process can occur when students return home. After internalising Western ideas of self-expression, emotional openness, and individual autonomy, returnees may find themselves readjusting to collectivist expectations that prioritise family consultation, discretion, and relational harmony (Chaliawala, 2026). While Berry's model helps explain the psychological challenges of navigating multiple cultural contexts, it does not fully capture how individuals integrate these experiences into their evolving sense of self. Rather, this process reflects what Bhatia (2018) describes as cultural hybridity, where multiple cultural frameworks coexist rather than fully replace one another. For returnees, these frameworks may feel both enabling and unsettling, shaping how distress is interpreted, communicated, or deliberately left unspoken.

Identity negotiation theory further extends this perspective by explaining how individuals actively manage their sense of self within changing social and cultural contexts (Swann, 2005). Individuals are motivated to seek recognition that validates their internal self-concept while also striving for acceptance and belonging within their social environments (Swann, 2012; Ting-Toomey, 2017). For AISs returning home, this dynamic can generate subtle yet powerful tensions. Exposure to psychological norms in the United Kingdom, including greater openness around emotional expression and self-reflection, may influence how distress is understood and articulated. Being perceived as “Western-educated” can therefore carry symbolic value while also inviting scrutiny, particularly if attitudes are viewed as overly individualistic or emotionally transparent within more collectivist cultural contexts. Research on international student adjustment consistently highlights the importance of belonging and identity validation for psychological wellbeing during periods of cultural transition (Alshammari et al., 2023; Ziaian et al., 2021). In response, returnees may engage in strategic identity management, selectively disclosing emotional difficulties, modulating psychological language, or maintaining silence to align with culturally resonant norms. Mental health thus becomes not only an internal psychological state but also part of an ongoing relational process through which individuals negotiate how they understand themselves and how they are expected to present themselves within their home communities.

## Research questions

This study was guided by the following research questions:

1. How do AISs interpret their mental health experiences following reintegration into Algeria after studying in the United Kingdom?
2. How do returning students understand and negotiate disclosure and help-seeking within their home cultural and linguistic contexts?
3. How do they describe their coping strategies post-return, and what meanings do they attribute to these approaches?

## Method

### Design

This study adopted a qualitative research design informed by an interpretivist epistemological perspective, which recognises that experiences of mental health are socially, culturally, and linguistically constructed rather than fixed or universal phenomena. From an interpretivist standpoint, meanings of distress, wellbeing, and help-seeking are shaped through social interaction, cultural norms, and shared systems of meaning (Willig, 2019). This perspective was particularly appropriate given the study’s focus on understanding how returning international students interpret and negotiate mental health following reintegration into their home cultural context. A qualitative approach was selected to enable an in-depth exploration of participants’ subjective experiences and meaning-making processes. Reintegration after studying abroad represents a complex life transition involving identity renegotiation, shifting cultural expectations, and changing social roles. Such processes are contextually embedded and often emotionally sensitive, making them difficult to capture through quantitative approaches. Qualitative research is well suited to examining how individuals understand and articulate lived experiences within their social and cultural environments (Creswell & Poth, 2016).

### Participants and recruitment

Participants were AISs who had previously completed a period of study in the United Kingdom and had since returned to Algeria. A purposive sampling strategy was used to recruit individuals who could provide rich, relevant, and diverse accounts of post-return experiences. Eligibility criteria included being an Algerian national, having studied in the United Kingdom, and having returned to Algeria following completion of their studies. Participants were recruited through online networks, alumni groups, and snowball sampling. This approach enabled access to individuals who had shared experiences of international study and reintegration while ensuring variation in gender, field of study, and time since return. Sampling continued until sufficient depth and richness of data were achieved. Sampling was guided by the principle of information power, with sufficient depth achieved to address the research aims (Roberts et al., 2026).

### Participants

Twelve Algerian International Student (AIS) returnees participated in the study. Participants were aged between 30 and 36 years (M = 32.33); ten were female and two were male. All were native Algerian Arabic speakers who had completed postgraduate study in the United Kingdom and had returned to live in Algeria between six months and five years prior to data collection (Table I). The relatively narrow age range reflected the characteristics of the eligible postgraduate returnees reached through the recruitment networks rather than a predetermined sampling restriction. Participants were therefore at a broadly similar life and career stage, which may have shaped their experiences of reintegration differently from those of younger undergraduate returnees.

Participants were recruited between July 2022 and February 2023 using snowball sampling through academic networks, social media groups for Algerian graduates, and professional contacts. This approach was appropriate given the relatively small and hard-to-reach population of AISs who had returned home and the cultural sensitivity surrounding mental health disclosure. Several individuals who initially expressed interest later withdrew due to concerns about discussing mental health in relation to employment or professional reputation, reflecting ongoing stigma in the local context. Inclusion criteria required participants to be over 18 years old, fluent in English, to have completed postgraduate study in the UK, and to have returned to Algeria within the specified timeframe of six months to five years. Individuals who completed their studies remotely from Algeria or who were currently receiving professional mental health treatment were excluded. In line with interpretative phenomenological and reflexive thematic approaches, sample size was guided by depth and richness of analysis rather than representativeness. The final sample provided a rich and information-powerful dataset suitable for in-depth qualitative analysis. The predominance of female participants reflected the composition of those who responded to the recruitment strategy and should be considered when interpreting the findings, particularly because gender may influence experiences of stigma, disclosure, and help-seeking.

**Table I.** Demographic characteristics of participants (*N* = 12).

No.	Pseudonym	Age	Gender	Place of Study	Duration Abroad	
1	Amelia	33	Female	England	4 years	
2	Liliyana	32	Female	England	5 years	
3	Mathew	30	Male	England	5 years	
4	Lilliyan	33	Female	England	4 years	
5	Emmie	32	Female	Scotland	6 years	
6	Lea	34	Female	England	5 years	
7	Tara	32	Female	England	2 years	
8	Aicha	35	Female	England	4 years	
9	Davie	30	Male	England	5 years	
10	Rinda	30	Female	England	4 years	
11	Katie	33	Female	England	5 years	
12	Abbie	34	Female	England	4 years	

Note: All participants were AIS returnees who had completed postgraduate study in the United Kingdom and returned to Algeria.

### Data collection

Data were collected through semi-structured, in-depth interviews conducted online. An interview guide was developed based on the research questions and prior findings from the broader project. Questions explored experiences of reintegration, understandings of mental health, disclosure practices, help-seeking, and coping following return to Algeria. Interviews were conducted in Arabic, French or English according to participant preference and lasted between 24 and 80 minutes (M = 48 minutes, 20 seconds). All interviews were audio-recorded with consent and transcribed verbatim prior to analysis. Where interviews were conducted in Arabic or French, transcripts were translated into English for analysis. Translation was undertaken by the first author, who is fluent in Arabic, French, and English. Translation was approached as an interpretative process, with careful attention paid to preserving the meaning, cultural context, and nuances of participants' accounts rather than producing literal word-for-word translations. To support meaning equivalence across languages, a sample of translated quotations was independently spot-checked against the original Arabic and French transcripts by a research assistant who was a doctoral student fluent in both languages, and ambiguous phrases or translation decisions were discussed and resolved collaboratively during supervision meetings.

### Data analysis

Data were analysed using reflexive thematic analysis (RTA), informed by an interpretative phenomenological sensibility. Reflexive thematic analysis is a theoretically flexible method that supports rich, interpretative engagement with participants’ lived experiences while recognising the active role of the researcher in knowledge production (Braun & Clarke, 2006, 2019, 2021). This approach was selected because it allows for the exploration of shared patterns of meaning across a dataset while remaining attentive to nuance, context, and subjectivity. The analytic process followed the six-phase approach outlined by Braun and Clarke, beginning with repeated reading of transcripts to support deep familiarisation with the data. Initial codes were then generated to capture meaningful features of participants’ accounts. These codes were iteratively organised into themes, which were reviewed, refined, and defined through sustained engagement with the dataset and ongoing reflexive dialogue within the research team.

The analysis was informed by an interpretative phenomenological orientation, which prioritises the exploration of how individuals make sense of their lived experiences (Smith, 2017). Rather than undertaking a full Interpretative Phenomenological Analysis (IPA) (Nizza et al., 2021), interpretative phenomenological principles were used to sensitise the analysis to participants' meaning-making, lived experiences, and sociocultural contexts. The primary analytic method remained reflexive thematic analysis, with themes developed across the dataset rather than through detailed idiographic case analysis. This orientation guided a focus on meaning-making, context, and the subjective interpretation of experience. Combining an IPA-informed lens with reflexive thematic analysis is increasingly recognised as appropriate where researchers aim to retain idiographic sensitivity while developing themes that capture shared patterns across participants (Nigbur & Chatfield, 2025; Spiers & Riley, 2019). This approach enabled the analysis to move iteratively between individual accounts and cross-case patterns, supporting both depth and interpretative richness.

### Reflexivity

Reflexivity was an ongoing component of the research process. The lead researcher (NB) occupied an insider position as an Algerian international student who had studied in the United Kingdom and returned home. This shared cultural and experiential background facilitated rapport with participants and supported nuanced understanding of culturally embedded meanings, language use, and contextual references within the data. At the same time, this positionality required ongoing reflexive engagement to consider how personal experiences, assumptions, and prior knowledge might shape data interpretation and analytic decision-making. NB is multilingual and attentive to how emotional distress may be expressed differently across Arabic, French, and English, which supported sensitivity to linguistic nuance and the ways participants navigated mental health meanings across languages.

To support transparency and critical self-awareness, the researcher maintained reflexive notes throughout data collection and analysis, documenting emotional responses, emerging interpretations, and potential assumptions (Shufutinsky, 2020). Regular discussions with the supervisory team (NC and SK), who were positioned as cultural and academic outsiders to the Algerian context, provided opportunities for reflexive dialogue and analytic challenge. These discussions supported reflexive questioning of interpretations, encouraged consideration of alternative explanations, and helped balance insider insight with critical distance. This combination of insider and outsider perspectives contributed to a reflexive and collaborative analytic process aimed at enhancing credibility, transparency, and interpretative depth.

### Ethical considerations

This study received ethical approval from the University Research Ethics Committee (Ref: 71.14.04. 2022). All participants received an information sheet and provided informed consent prior to participation. Interviews followed a guide, and participants were reminded that they could pause, or withdraw at any time if they feel uncomfortable. Participants were assured of confidentiality and anonymity, and identifying details were removed from transcripts. Given the sensitive nature of mental health discussions, participants were also provided with information about available support services available in Algeria.

## Results

This study explored how AISs made sense of their mental health experiences after returning to Algeria from the UK. Using an interpretative phenomenological sensibility alongside reflexive thematic analysis, five interconnected themes were developed that illuminate how participants’ understandings of mental health evolved, how disclosure and help-seeking were negotiated, and how coping was shaped by reintegration into a familiar yet altered cultural context. Together, the themes highlight the interplay between cultural meaning systems, identity negotiation, and linguistic resources in shaping post-return wellbeing. The five themes were: (1) Shifting perceptions and evolving understandings, (2) Struggles of return, (3) Silent struggles, (4) Coping with the unfamiliar in familiar surroundings, and (5) Linguistic duality in mental health narratives (see Table II).

**Table II.** Overview of themes and illustrative quotations.

Theme	Description	Example Quotation
Shifting perceptions and evolving understandings	Exposure to UK mental health discourse increased awareness and legitimised distress.	“Before I had no awareness of it… now I feel aware.”
Struggles of return	Reintegration involved reverse culture shock and identity renegotiation.	“You don’t know whether your family would accept the new you.”
Silent struggles	Disclosure was shaped by stigma and fear of judgement.	“The fear of being alienated can stop you from sharing.”
Coping with the unfamiliar in familiar surroundings	Participants developed new coping strategies and selective disclosure.	“I pretended I was okay… inside I felt disconnected.”
Linguistic duality in mental health narratives	Language shaped how distress was understood and communicated.	“I feel like I am translating myself, not just the language.”

Theme 1: Shifting perceptions and evolving understandings of mental health.

Participants described how living in the UK expanded their awareness of mental health and introduced new ways of naming, interpreting, and legitimising emotional distress. For many, mental health was not previously framed as a distinct topic of discussion in Algeria, even when distress was present. Instead, awareness appeared to emerge through lived experience, exposure to institutional supports, and increased access to psychological language that made internal states more recognisable and socially intelligible. Several participants described a movement from conceptual distance to a more embodied conviction that mental health “really exists,” rooted in experience rather than abstract knowledge. As Katie reflected:

“At the beginning... I wouldn't say it didn't make sense to me, just the expression I would say of mental health before, but now I feel it really exists. So, I understand it more. Before I had no awareness of it. But now I feel like I'm aware of it. I think because of my experience. So that's why I became aware.” (Katie)

Here, “awareness” is presented as something that developed through personal encounter, suggesting that mental health became real and meaningful when it demanded recognition in her own life.

Participants also located limited recognition of mental health within institutional and everyday contexts in Algeria, distinguishing between the presence of distress and the absence of a shared framework for understanding it. Lilyana noted:

“Speaking about mental health when I was a master student or bachelor student; I mean we've never really had a discussion about mental health... definitely, we were experiencing mental health issues, but we have never really pinpointed them as mental health. But the moment I went to the UK, the first thing I was introduced to was university mental health campus… for the first two years I didn’t really pay attention... because we already don’t have mental health services at the level of universities in Algeria.” (Lilyana)

Her account highlights how new systems of support may take time to feel relevant, particularly when they are unfamiliar within one’s prior cultural and institutional experience.

A recurring shift concerned the power of naming. Participants described how diagnostic and psychological vocabulary provided clarity and legitimacy, while Algerian framings were often experienced as moralised or dismissive. Aicha explained:

“When I was in the UK I could name things because I experienced things related to mental health like anxiety, like distress. It's only then that I could have names… in the West I felt that every experience has a name and a diagnosis, whereas here in Algeria some things are just what we call someone not being as tough as they should be, when in fact it’s a mental health condition.” (Aicha)

Naming, here, functions as both understanding and validation, while the alternative framing (“not being as tough”) positions distress as personal weakness rather than a legitimate health experience.

Theme 2: Struggles of return and mental health in familiar territory.

Returning home after years of study abroad was rarely described as straightforward or comforting. Instead, participants portrayed reintegration as emotionally layered, disorienting, and marked by reverse culture shock. Home was experienced as both familiar and unfamiliar, requiring participants to adapt not only to their environment but also to the changed selves they brought back with them. participants frequently described the need to “adapt” and “readapt,” reflecting a cyclical process rather than a single transition. As Mathew explained:

“This is really representative; the main challenge would be to adapt or readapt to the Algerian context because four or five years studying abroad definitely changes how you see things. Readapting is the key challenge because taking mental health into account, you no longer believe in traditions like possession, jinn, or black magic before science. That new way of looking at the world doesn’t always match the Algerian reality, which is challenging.” (Mathew)

Mathew's account illustrates that reintegration extended beyond practical adjustment. Returning home required participants to reconcile new ways of understanding themselves and the world with cultural expectations that no longer felt entirely familiar.

“We had different habits, maybe a different lifestyle, and you don't know whether your family, friends, or wider social circle will accept the new you or not.” (Mathew)

Here, reintegration is presented as both a personal and relational challenge. Returning home required renegotiating belonging and seeking acceptance from family and social networks.

Uncertainty about the future was another prominent feature of reintegration. Participants described the end of structured academic life abroad as destabilising and emotionally demanding. Aicha reflected:

“I think that the challenge is that he might face would be about I think thinking about the future. It’s true that he will go back home. And then what; what’s next; the challenge now is to start over a new cycle or new routine and the biggest challenge is to not compare between UK and Algeria after having different life for four or five years.” (Aicha)

Her repeated questioning “and then what; what’s next” captures the ambiguity of return, where homecoming did not provide the anticipated sense of resolution. Participants also described how returning home altered their perception of familiar relationships. Abbie explained:

“After I returned home my happiness fade away as I started seeing a different reality or I would say different world meaning different from the one I used to be in which is UK. It felt weird for me to see my family differently than I used to see them and to struggle from those differences because I didn’t used to see them as in mental health expressions; when my mom is not feeling well I ask her to go out and change her mood and to avoid being depressed she was against this term almost all family were as they don’t believe in it and as in mental health is something normal and we all go through that and that honestly made me suffer and regret my return at some point.” (Abbie)

Her account highlights how changes in perception reshaped relationships and generated feelings of regret and emotional distance. Participants consistently described a sense of misunderstanding and social distance following their return. Lilyana summarised this experience succinctly:

“I think the key challenges the first one is misunderstanding I mean the surrounding whatever friends, or family because you know you are different than them now and that you have been changed after leaving the country for about five years.” (Lilyana)

Similarly, Mathew described how comparison between cultural contexts became emotionally uncomfortable:

“I was not seeing these things before and being far away for some time and returning home made me always compare and that comparison especially speaking about culture and privacy thing, or collectivism versus individualism was something very clear for me, and that was not a comfortable feeling to live with.” (Mathew)

These accounts illustrate how cultural comparison, while increasing awareness, also intensified feelings of alienation. Finally, participants highlighted the challenge of adjusting to the pace and rhythm of everyday life after returning home. Emmie reflected:

“In terms of the challenges, I can only imagine one of the main issues would be a question of adjustment. So, adjusting again to the rhythm, you know, to the pace, to the sort of the kind of life you know the Algerian life. Also, I would say, in terms of the lifestyle, you know, because the lifestyle that an Algerian student might have in the UK would definitely be different from the situation going back home so that sort of change can also be challenging which definitely affected our mental health.” (Emmie)

Across accounts, returning home was framed as an ongoing negotiation between past and present selves, between familiarity and change. Reintegration emerged as a continuous process of rebuilding belonging, renegotiating relationships, and reconstructing stability within a context that felt both intimate and transformed.

Theme 3: Silent struggles and barriers to help-seeking.

After describing the emotional challenges of reintegration, participants revealed another layer of difficulty: the challenge of seeking psychological support in a cultural context where disclosure was often experienced as risky. Help-seeking was shaped not only by access to services but by fears of judgement, social exclusion, and misunderstanding. Speaking about distress was described as crossing invisible cultural boundaries and risking one’s social identity. A central concern was fear of social alienation. Participants described how disclosure could threaten belonging within family and community networks. Lea explained:

“When you are part of a group, there is always the fear of being alienated. That fear can stop you from sharing or disclosing your experiences because others may find them strange or even unacceptable for religious, social, or cultural reasons. If your experiences don't reflect what is expected of you as an Algerian, Arab, and Muslim...” (Lea)

Lea's account illustrates that disclosure was not simply about revealing emotional distress but about protecting social identity and preserving belonging within valued social groups.

“…then you risk being rejected from the groups you belong to.” (Lea)

Here, disclosure is framed as a social risk that may lead to rejection from valued groups, highlighting the collective dimension of mental health stigma.

Here, disclosure is framed as a social risk that may lead to rejection from valued groups, highlighting the collective dimension of mental health stigma. Participants also described how cultural expectations shaped what it meant to be an “acceptable” member of society. Emotional vulnerability was often perceived as incompatible with social ideals of strength and composure. Silence therefore became a protective strategy that safeguarded both personal and family reputation. Religion was described as both a source of comfort and a barrier to seeking professional help. Mental distress could be interpreted as a spiritual weakness rather than a psychological concern. Mathew reflected:

“As I said earlier, example yeah if I tell someone I'm really depressed, they might say you're not brave enough, spiritual enough maybe for some people, yes or something people, they would stop them from seeking the help they need.” (Mathew)

This framing positioned help-seeking as unnecessary or even morally questionable, reinforcing self-reliance and emotional endurance. Fear of judgement and misunderstanding further discouraged disclosure. Participants worried that their distress would be trivialised or misinterpreted. Abbie explained:

“I think a lot of reasons would be included, fear of being judged, or fear of being misunderstood, or also as I said before avoiding people who would believe and think ah you are just trying to avoid the black eye by showing the negative things, it also can be that he cannot find someone who can understand him and support him.” (Abbie)

Her account highlights the emotional labour involved in anticipating negative reactions before disclosure even occurs. Trust emerged as a crucial factor shaping decisions about professional help-seeking. Even when services were available, participants questioned whether professionals would truly understand their experiences. Katie described how she ultimately decided not to seek therapy:

“I started looking for I like on social media and everything, and even convinced to seek professional help. Then she advised not to go. Why? Because she said, oh, they may not understand really your situation, because maybe they did not experience what you experienced so maybe they can’t help you, really. And I actually, I didn't go to be honest.” (Katie)

Similarly, Rinda emphasised the importance of emotional trust over formal qualifications:

“I’ve considered seeking professional help here, I just, I don’t think I’ve ever been able to find someone who I felt was qualified enough to be able to help me with my issue.” (Rinda)

In these accounts, perceived understanding and emotional resonance were central to whether professional support felt legitimate. Participants also described practical and financial barriers that reinforced hesitation. Lea explained:

“The first thing that comes to my mind is the cost. If you're not working and develop severe mental health difficulties, professional help isn't free, so the first challenge is simply being able to pay for it.” (Lea)

Alongside financial barriers, participants described limited public awareness of mental health, which further discouraged professional help-seeking and reinforced stigma.

“Even if you ask your family, some may think professional help is unnecessary or ask whether you're ‘crazy’. Many people are not psychologically aware of how important mental health is, and some cannot distinguish between a psychologist and a psychiatrist. These are some of the biggest challenges.” (Lea)

Together, these accounts illustrate how silence surrounding mental health is sustained by an interwoven set of cultural, emotional, and structural factors. Seeking help was not simply an individual decision but a complex negotiation shaped by social belonging, trust, stigma, and access.

Theme 4: Coping with the unfamiliar in familiar surroundings.

Participants described coping with reintegration as an ongoing psychological process shaped by feelings of estrangement, identity change, and the need to rebuild stability. Returning home was often expected to bring comfort and familiarity, yet many experienced subtle disconnection from people, routines, and environments that once felt central to their identity. Coping therefore involved renegotiating belonging while making sense of personal growth and change. Several participants described feeling like strangers within familiar spaces. Rania explained:

“When I came back, I felt like I didn’t belong in my own house. The same walls, same family, but something was off. I couldn’t relate to the way they spoke or what they talked about anymore. They were all in their rhythm, and I was just… watching.” (Rania)

Her account illustrates how reintegration could disrupt previously taken-for-granted feelings of belonging and emotional connection. Participants also described tension between their changed identities and others’ expectations that they would return unchanged. Amine reflected:

“My parents were happy to see me, of course, but I realised they were expecting the same old me. They wanted the same routines, same jokes, same way of thinking. But I had changed. I couldn’t just go back to being that version of me, and it made conversations feel forced.” (Amine)

This expectation to resume a previous identity created emotional strain and required ongoing negotiation within relationships. For some participants, coping involved reframing change and accepting difference as a natural outcome of growth. Mathew described this process:

“I struggled at the beginning as I was looking for someone who can understand my feelings. I was like a refugee searching for ways and sources to cope. But after some time, I learnt about differences. Though it was my homeland and my mother culture, I acknowledged that people after some time in their life—including Covid-19—are different now, even within the same place they were born in. So doing that started to calm me down and to feel, yeah actually it’s fine, as I acknowledge that even my family sees me differently.” (Mathew)

Here, coping is framed as a process of meaning-making and acceptance rather than a simple return to previous patterns of belonging. Other participants described initially coping by performing normally while gradually adjusting internally. Sara explained:

“At first, I used to pretend I was okay and act like before. I laughed, went out, and joined family events, but inside I felt disconnected. My mindset didn’t match theirs anymore. It took time for me to stop pretending and to just accept that maybe I’m different now.” (Sara)

This account highlights the social dimension of coping, where outward behaviour and internal experience may diverge during the adjustment process. Some participants adopted practical and activity-based coping strategies to rebuild structure and stability. Yasmina described:

“I decided to keep myself busy. I went back to work quickly, joined some courses, and focused on my goals. That helped me a lot. The more I stayed active, the less I thought about how different everything felt.” (Yasmina)

Maintaining routines and engaging in purposeful activity provided a sense of agency and continuity following return. Across accounts, coping was shaped by a gradual redefinition of home as a fluid and evolving concept rather than a fixed place. Participants described learning to harmonise personal growth with collective belonging through reflection, negotiation, and acceptance. Coping therefore involved both emotional and social adaptation, highlighting the ongoing nature of reintegration.

Theme 5: Linguistic duality in mental health narratives.

Language emerged as a central resource through which participants made sense of their mental health after returning home. Having spent years learning to discuss wellbeing in English, many described difficulty expressing the same experiences in Arabic. This created a form of linguistic and emotional tension: the language that enabled them to understand their mental health was not always the language available in everyday life at home. Participants therefore navigated mental health through bilingual expression, using language as a tool for emotional regulation, identity negotiation, and communication. Participants frequently described how mental health vocabulary carried different emotional and social meanings across languages. Words that felt neutral in English were experienced as heavy, extreme, or morally charged in Arabic. Mathew explained:

“When I talk about depression in Arabic, it doesn’t sound the same. The word feels heavier, and people understand it differently. In English I can say it easily, but in Arabic it feels like I’m saying something extreme or shameful, like I’m saying I’m mad, not just sad.” (Mathew)

This contrast illustrates how language shapes both communication and the perceived legitimacy of distress. Speaking about mental health in Arabic was often associated with stigma and moral judgement, making openness more difficult. For many participants, English provided a form of emotional distance that made it easier to articulate distress. Speaking in English was described as less exposing and less emotionally intense. Lilyan reflected:

“I think I prefer to speak in English when I talk about my mental health. It feels like I can say things that in Arabic would sound too strong or too personal. English makes it easier to speak without feeling too vulnerable.” (Lilyan)

Participants also described the strain of translating mental health concepts across languages and cultural contexts. Rinda explained:

“Sometimes I start explaining something I learned in the UK, like anxiety or burnout, but I stop halfway because I can’t find the right word, or because I know they will not get it the same way. I feel like I am translating myself, not just the language.” (Rinda)

The phrase “translating myself” captures the emotional and identity-related labour involved in communicating across linguistic and cultural frameworks. While English provided distance and protection, participants also described their mother tongue as more emotionally expressive and authentic. Amelia explained:

“I would say I have done this for two major reasons; one is that it is more expressive to use my dialect, and second is that it is more direct to the point and doesn’t need further explanation, unlike in English. I feel I need to be more careful to choose my words.” (Amelia)

Similarly, Lilyana described an instinctive pull toward her dialect:

“I didn’t really do that on purpose, but I felt the need to express my point of view in my dialect as I felt it’s more expressive.” (Lilyana)

Despite these tensions, many participants framed bilingualism as a resource that supported emotional adaptation and identity integration. Davie explained:

“At first it was hard because I couldn’t talk in the same way, but then I realised I can use both languages; I can think in English and explain in Arabic, and that helps me to make sense of what I feel. It’s not perfect, but it works for me.” (Davie)

Across accounts, language functioned as both a mirror of identity and a coping resource. English offered conceptual clarity and emotional distance, while Arabic and dialect provided authenticity and belonging. Navigating between languages allowed participants to reconstruct continuity between their experiences abroad and their lives at home, highlighting the role of bilingualism in supporting psychological adaptation after return.

## Discussion

This study explored how AISs made sense of mental health after returning to Algeria following their study in the UK, focusing on evolving understandings of distress, reintegration challenges, disclosure and help-seeking barriers, coping strategies, and the role of language. Across the five themes, participants' accounts demonstrate that reintegration was not experienced as a simple “return” to a stable home identity. Instead, it involved an ongoing negotiation between personal transformation and contextual continuity, as mental health literacy gained abroad interacted with culturally embedded norms, relational expectations, and linguistic constraints. Participants described shifts in the way mental health was recognised and named (Theme 1), followed by a re-entry phase characterised by reverse culture shock and emotional disorientation despite physical familiarity (Theme 2). These findings suggest that distress arose not because participants expected Algeria to change, but because returning home made differences between their transformed sense of self and their familiar social environment increasingly visible. This aligns with work on re-entry adjustment and reverse culture shock showing that returning home can be destabilising precisely because it is assumed to be easy and because both person and context have changed, often in different ways (Fanari et al., 2021; Ward et al., 2000). Participants' accounts also indicate that reintegration is an active process of psychological adaptation, rather than a passive readjustment to pre-existing roles, in which belonging must be continually renegotiated rather than automatically restored (Winkel et al., 2022).

A central thread running through Themes 1 and 2 was the role of comparison as a cognitive and emotional sense-making practice. Participants made sense of reintegration by comparing Algeria and the UK, using these contrasting experiences to interpret cultural norms, mental health discourses, and changes in selfhood. Comparison has been described as a core social-cognitive process that supports interpretation and decision-making by relating new experiences to existing knowledge (Kedia et al., 2014). Unlike previous work, however, participants were comparing two cultural contexts they knew intimately through lived experience rather than navigating an unfamiliar setting. This enabled them to recognise what had changed in themselves, what remained stable in their home environment, and how understandings of mental health differed across contexts. Participants' narratives also reflect cognitive dissonance processes (Dilakshini & Kumar, 2020), whereby discomfort emerged when newly internalised psychological frameworks conflicted with prevailing interpretations at home, prompting efforts to reconcile these tensions through reappraisal, selective disclosure, or changes in expectations (Ellis & Cromby, 2009). Rather than simply evaluating one context against another, participants used comparison to reconcile evolving identities, mental health understandings, and cultural expectations. Comparison therefore functioned as a process of psychological sense-making that helped to integrate two coexisting systems of meaning following return. Themes 3 and 4 further show that disclosure and help-seeking were shaped by social risk rather than need alone. Participants described fear of judgement, misunderstanding, and social rejection, highlighting how mental health expression could threaten belonging within family and community networks. These accounts are consistent with stigma theory, in which disclosure becomes costly because it risks negative moral and social categorisation (Corrigan & Matthews, 2003), and with evidence that re-entry experiences are profoundly relational, involving uncertainty about whether the “new self” will be accepted by important others (Hedegaard & Hugo, 2022). Social Identity Theory offers a useful lens here: participants’ narratives suggest that cultural, religious, and gendered expectations regulated emotional expression and help-seeking, with deviation from shared norms perceived as a risk to group membership and identity legitimacy (Tajfel & Turner, 1986). Religious framings could position distress as a test of faith or a marker of insufficient spirituality, reinforcing silence and discouraging professional help-seeking (Hardy et al., 2013). These findings mirror broader evidence in collectivist and religiously embedded contexts where stigma, concerns about reputation, and moralised interpretations reduce openness and formal service use (Jacob et al., 2025; Ran et al., 2021).

Participants' accounts also point to substantial practical and systemic barriers to professional help-seeking, including financial constraints, limited service availability, and distrust of professional competence. Concerns that professionals would not fully understand their experiences align with research demonstrating that perceived legitimacy, cultural responsiveness, and trust influence engagement with mental health services as much as service availability (Opia & Matthew, 2025; Skinner-Osei & Osei, 2024). These findings suggest that reintegration support should extend beyond awareness-raising to include culturally responsive counselling pathways, efforts to reduce stigma, and services that foster trust, relational safety, and confidence in professional care. Coping strategies (Theme 4) reflected both adaptation and emotional compromise. Participants described keeping busy, re-establishing routines, selectively disclosing distress, “pretending” to fit in, and seeking support from others with shared experiences. The value of peer connection and being understood by fellow returnees aligns with social support theory, which emphasises the protective role of validation and shared meaning in reducing isolation and distress (Ruihua et al., 2025). At the same time, several accounts imply that acceptance of difference was not always benign or relieving; for some, “acceptance” required suppressing internal change to maintain harmony, suggesting an emotional cost to belonging. In this study, coping often involved managing this trade-off while maintaining connection without erasing personal transformation.

A distinctive contribution of this research is the demonstration that reintegration is not only cultural and psychological but also linguistic (Theme 5). Participants described using English and Arabic/dialect differently to manage emotional exposure, communicate distress, and preserve belonging. This supports bilingualism research suggesting that a second language can provide emotional distance that makes disclosure feel safer, while a first language may carry stronger affective resonance and social meaning (Toivo et al., 2024). Participants' narratives further suggest that language choice was not simply a matter of preference but served as a coping strategy. English enabled conceptual clarity and reduced vulnerability, while Arabic and dialect supported authenticity, cultural grounding, and relational connection. Difficulties translating mental health terms and the moral weight carried by certain Arabic labels illustrate how language can intensify stigma and constrain empathy, shaping whether distress is met with understanding, dismissal, or moral judgement (Slewa-Younan et al., 2022). Drawing on complementary bilingualism, moving between languages may help returnees bridge cultural worlds and negotiate how much emotion to reveal or contain (Grosjean, 2021). These findings suggest that post-return wellbeing is influenced not only by what individuals experience, but also by whether they can communicate distress in ways that feel both authentic and culturally meaningful.

The practical implications extend beyond the study-abroad period. Universities should prepare students for reintegration before they return home by providing structured re-entry programmes, psychoeducation on reverse culture shock, and opportunities to connect with previous returnees. Policymakers and mental health services should complement these initiatives by developing culturally responsive counselling pathways, strengthening mental health literacy, reducing stigma, and fostering trust in professional care (Cogan et al., 2024). Peer-led returnee networks may be particularly valuable by providing psychologically safe spaces in which experiences can be shared without the social costs often associated with disclosure within home communities (Cogan et al., 2022; Egmose et al., 2024).

Finally, language should be recognised as more than a communication tool. It forms part of identity negotiation, emotional regulation, and help-seeking, shaping how returnees make sense of distress, access support, and maintain belonging. Together, these findings demonstrate that reintegration is a distinct phase of international mobility that requires culturally and linguistically responsive support extending beyond the period of study abroad.

### Strengths, limitations and future directions

A key strength of this study lies in its in-depth qualitative exploration of the post-return mental health experiences of Algerian international students, a population that remains underrepresented in the literature. By focusing on reintegration rather than adjustment during study abroad, the study addresses a significant gap in international student mental health research. The use of rich, first-person narratives enabled rich insights into how participants made sense of distress, identity change, disclosure, and coping within culturally and religiously embedded contexts. Retaining participants' own words allowed the analysis to remain grounded in lived experience and strengthened the credibility and authenticity of the findings.

Another strength is the study's attention to language as a psychological and cultural resource. By examining how bilingualism shaped emotional expression, disclosure, and meaning-making, the study extends existing work on mental health literacy and cross-cultural psychology. This focus offers an original contribution by highlighting the linguistic dimension of reintegration, which is often overlooked in mental health research on international students. The findings therefore contribute to a growing body of global mental health research emphasising the importance of culturally and linguistically situated understandings of wellbeing.

Several limitations should also be acknowledged. The sample consisted of Algerian students who had studied in the UK for extended periods, which may limit transferability to students returning from other host countries or with shorter study durations. The relatively narrow age range and predominance of female participants may also influence the transferability of the findings, as reintegration experiences may differ across demographic groups. Participants were also reflecting retrospectively on their experiences, meaning that their accounts may have been influenced by memory, current circumstances, and ongoing processes of meaning-making. As with all qualitative research, the findings are not intended to be statistically generalisable but rather to offer analytic insight into processes that may resonate across similar contexts.

These limitations also highlight important directions for future research. Longitudinal studies following students before departure, during study abroad, and after return would provide valuable insight into how mental health literacy, identity, and coping evolve over time. Comparative research across different cultural, linguistic, and religious contexts could further illuminate how reintegration experiences vary globally. Future research should also prioritise the development and evaluation of reintegration-focused interventions, including structured re-entry programmes, peer-support initiatives, culturally adapted psychoeducation, and bilingual mental health resources designed specifically to support returning international students. Co-creation methodologies involving translation and cultural localisation, as demonstrated in recent digital mental health intervention research with other high-stress populations (Cogan et al., 2026), offer a promising model for developing linguistically and culturally responsive digital support tools for AIS returnees, ensuring that content is co-produced with the community it is intended to serve rather than adapted post hoc from Western frameworks.

## Conclusion

This study explored how Algerian international students experienced mental health and reintegration after returning home, revealing reintegration as an emotionally complex and ongoing process rather than a simple return to familiarity. Participants' accounts demonstrated that mental health, identity, coping, and disclosure were shaped by cultural norms, relational expectations, religious meanings, and linguistic constraints. The findings highlight the need for support that extends beyond the study-abroad period. Universities should prepare students for reintegration through re-entry support and peer connections, while mental health services should provide culturally responsive care that addresses stigma, trust, and the cultural and religious meanings attached to distress. By centring the voices of returning students, this study advances understanding of reintegration as a distinct phase of international mobility with important implications for global mental health. Recognising and supporting this transition is essential to promoting long-term wellbeing among internationally mobile students.

## Data Availability

The data that support the findings of this study are not publicly available due to ethical and confidentiality restrictions. The qualitative interview data contain potentially identifiable personal narratives and cannot be shared in full. De-identified excerpts are presented within the article. Additional methodological information may be available from the corresponding author upon reasonable request.
